# Assessing nonresponse bias in a 30-year study of gulf war and gulf era veterans

**DOI:** 10.1186/s12874-025-02761-5

**Published:** 2026-01-10

**Authors:** Joseph Gasper, Wendy Van de Kerckhove, Talia Spark, James McCall, Carly Mihovich, Heather Hammer, Aaron Schneiderman, Michele Madden, Erin K. Dursa

**Affiliations:** 1 Westat, 7501 Wisconsin Ave, Suite 1000E, Bethesda, MD 20814 USA; 2 Independent Consultant, Philadelphia, PA USA; 3 U.S. Department of Veterans Affairs, Office of Patient Care Services, Health Outcomes Military Exposures, 810 Vermont Ave NW, Washington, DC 20420 USA

**Keywords:** Veterans, Nonresponse bias, Cohort study, Longitudinal study, Weighting

## Abstract

**Background:**

Cohort studies of veterans are critical for understanding the long-term health effects of deployment and toxic exposures. However, longitudinal research is susceptible to attrition and potential nonresponse bias. The Gulf War Era Cohort Study (GWECS) is the largest and longest-running longitudinal cohort study of 1990–1991 Gulf War veterans. In this paper, we identify demographic and military service characteristics associated with patterns of response over time and examine the extent to which accounting for nonresponse bias in Wave 4, conducted more than 30 years after the Gulf War, might impact the estimates of health conditions.

**Method:**

Multivariate multinomial logistic regression analysis was used to identify demographic and military service characteristics associated with response patterns over time (always responder, current responder, past responder, never responder). To adjust for nonresponse at Wave 4, a search algorithm was used to identify predictors of response and form weighting class cells. The effectiveness of nonresponse adjustments was evaluated by (1) comparing estimates of demographic and military characteristics before and after weighting, (2) examining the correlation between the weighting classes and health outcomes, and (3) comparing early and late responders on health outcomes.

**Results:**

Wave 4 obtained a response rate of 47%, close to the 50% response rate obtained in Wave 3 over a decade earlier. Veterans most likely to respond to the survey over time and in Wave 4 were older, White, deployed, officers, and married in 1991. Weighting adjustments accounted for these differences and reduced bias in demographic and military characteristics, as well as in survey variables related to those characteristics. However, differences observed between early and late responders on alcohol and drug dependence suggest that these conditions may be underestimated.

**Conclusion:**

GWECS achieved a relatively high response rate in the most recent follow-up. Although differential response occurred, nonresponse adjustments effectively reduced bias in the key variables examined. This cohort continues to provide insight into the long-term health effects of Gulf War deployment, including cancer and chronic conditions, as the cohort ages.

**Supplementary Information:**

The online version contains supplementary material available at 10.1186/s12874-025-02761-5.

## Background

Cohort studies of veterans are extremely important for understanding the long-term effects of deployment and military environmental exposures on physical and mental health. However, longitudinal research is susceptible to attrition and potential nonresponse bias. Nonresponse bias is a function of both the overall nonresponse rate and the degree to which respondents and nonrespondents differ on the statistic of interest [1]. Several studies have shown that a low response rate may not necessarily produce substantial nonresponse bias if the differences are minimal or statistically negligible [2–4]. Nevertheless, nonresponse bias should be examined and addressed whenever any concerns about the representativeness of respondents arise.

Several factors are associated with a higher likelihood of follow-up response in general population studies, including female sex, older age, higher education attainment, and being married [5, 6]. Few studies of follow-up with military service members and veterans have published robust nonresponse analyses. The King’s Centre for Military Health Research Health and Wellbeing Cohort Study, a long-term study on the physical and psychological health of United Kingdom military personnel who deployed to Iraq or Afghanistan, found that responders at 20 years were more likely to be female, older age, reserve personnel at baseline, officer rank, had or currently served in the Royal Air Force (versus the Army), and reported symptoms of depression or anxiety [7]. The Millennium Cohort Study (MCS), which primarily includes post-9/11 veterans, found that consistent follow-up response over 15 years after entering the cohort was associated with higher educational attainment, being married, female sex, older age, and deployment [8]. Evidence for health-related predictors of response in the MCS is mixed. One analysis reported that life stressors, mental health conditions, and physical health diagnoses were associated with higher response, whereas an earlier analysis identified smoking, alcohol consumption, and depression as predictors of nonresponse [8, 9]. Nevertheless, nonresponse weighting in the MCS suggests that nonresponse did not substantially bias estimates of health outcomes [8, 9]. 

Most longitudinal studies report response comparisons at a single point in time, but response can be intermittent, with individuals responding to some waves but not to others. Several studies have classified participants into typologies based on their response histories, showing that intermittent responders tend to fall demographically between consistent responders and never responders [10]. This pattern suggests that longitudinal nonresponse exists along a continuum from severe to less severe [11, 12]. Understanding individual patterns of participation over time is important for two reasons. First, identifying who participates is essential for effectively and efficiently allocating data collection procedures and resources. In adaptive designs, recruitment and retention materials strategies can be tailored toward groups at highest risk for nonresponse, and resources can be directed to improving response rates among these groups [13]. Second, understanding nonresponse patterns can lead to insights into potential biases and help to test the validity of assumptions underlying study analyses.

The GWECS is a population-based, longitudinal cohort study of 15,000 veterans who deployed to the Persian Gulf between 1990 and 1991 (Gulf War veterans) and 15,000 Veterans who served elsewhere during the same period (Gulf Era veterans). Sponsored by the U.S. Department of Veterans Affairs (VA), the cohort has been surveyed in three previous waves of the GWECS, conducted in 1995, 2005, and 2012. The fourth wave of the study was conducted in 2024–2025 and included follow-up with all surviving, non-incarcerated members of the original cohort. The primary goal of the GWECS is to compare changes in self-reported health outcomes of Gulf War veterans with Gulf Era veterans over time, with a focus on chronic medical conditions, mental health conditions, functional impairment, and healthcare utilization. Additional objectives include characterizing the natural history of Gulf War illness as the population ages, assessing the impact of deployment-related exposures on long-term health, and examining overall health trajectories in both the deployed and non-deployed groups. The purpose of this study is to: (1) examine differences among groups with different response patterns to reveal potential sources of bias and inform recruitment and retention strategies (2) determine the extent to which adjustments for nonresponse bias in the fourth follow-up influence estimates of key health conditions. This study provides information that can inform study design, nonresponse adjustment, and analysis in other longitudinal studies of veterans.

## Methods

### Sampling

A permanent, population-based panel of 15,000 Gulf War veterans and 15,000 Gulf Era veterans was built using a stratified sampling design. Gulf War veterans were sampled from the 693,826 U.S. troops identified by the Defense Manpower Data Center (DMDC) as deployed to the 1990–1991 Gulf War Gulf War Era veterans were sampled from 800,690 persons (half of all personnel, those who were in the military service between September 1990 and May 1991) identified by DMDC as having served during that time but who did not deploy [14]. Both groups had representation from all service branches (Air Force, Army, Marines, Navy) [14]. To ensure adequate sampling of key subgroups, the sampling strategy for both the Gulf War and Gulf War Era groups included intentional oversampling of women. Women were oversampled to comprise 20% of the sample. The National Guard and reserves were oversampled to comprise about 27% and 33%, respectively. The remaining 40% of the sample consisted of active duty [14]. The veterans invited to participate in the 2024 follow-up study were all 26,580 living panel members of the original sample of 30,000 contacted in 1995.

### Data collection

The survey used a multimodal design that included web, mail, and computer-assisted telephone interviewing (CATI). The adaptive design strategy utilized veterans’ historical response patterns to tailor data collection protocols [15]. Veterans were classified into two groups based on historical response patterns. The majority of veterans received a sequential multimodal protocol, designed for veterans who responded to at least one of the previous waves of the survey. An invitation letter was mailed to this group, inviting them to complete the web survey using a unique personal identification number. Non-responders received a reminder postcard about the web survey and up to three mailed questionnaire packets. These packets contained a 16-page scannable structured health questionnaire, a preaddressed and prepaid return envelope, an informed consent form, a study brochure that included telephone numbers for study information, the VA Crisis Line number, and an information sheet that highlighted results from previous waves of the study. The invitation letter was sent to panel members in September 2024, accompanied by a reminder postcard mailed one week later. Three mailings of paper questionnaires occurred at 5, 8, and 13 weeks after the invitation letter. In November 2024, CATI calls were made to the veterans who had not responded to the web or paper survey. A smaller group of veterans who never responded to previous waves of the survey received a web-only protocol. Veterans in this group were mailed an invitation letter inviting them to complete the web survey, followed by a reminder postcard, three reminder letters, and a final reminder postcard. The VA Central Institutional Review Board and the Westat Institutional Review Board approved the study protocol and all documents. All data collection was completed by January 2025.

### Measures

#### Completion status

Completion status was determined based on the number of items completed on the survey. A complete questionnaire was defined as any questionnaire with ≥ 80% of the question items answered, and a partially complete questionnaire was defined as one with > 50% but < 80% of the question items answered. In total, 12,187 met the criteria for completed questionnaires, and 190 for partially completed questionnaires. Both partially and fully completed questionnaires were considered respondents.

To investigate response patterns, Wave 4 eligible respondents were categorized into four mutually exclusive groups based on their response patterns across waves. Always responders were defined as veterans who responded to all four waves. Never responders responded to none of the waves. Current responders responded to Wave 4 but did not respond to one or more previous waves, and past responders responded to one or more previous waves but did not respond to Wave 4.

#### Demographic and military service characteristics

Demographic and military service characteristics were obtained from military records. They included deployment status (deployed, nondeployed), age (17–25, 26–32, 33–39, 40 and older, unknown), race/ethnicity (White, Black, Hispanic, other), sex (male, female), marital status (married, single, other, unknown), service branch (Air Force, Army, Marine Corps, Navy), unit component (active duty, National Guard, reserves), and rank in 1991 (enlisted, officer, warrant officer). These variables were all measured in the sampling frame at the time of the 1990–1991 Gulf War.

#### Health outcomes

To evaluate the effectiveness of weighting in reducing potential nonresponse bias, several key outcomes were examined, including general health, posttraumatic stress disorder (PTSD) diagnosis, Gulf War illness (GWI) diagnosis, alcohol use, and cigarette use. Self-reported health was measured with a single question asking individuals to characterize their health as excellent, very good, good, fair, or poor [15]. Diagnosis of PTSD, GWI, alcohol or drug dependence, bipolar or manic depression, and Alzheimer’s disease or dementia were assessed in the survey by the following question: “Has a doctor or other health professional ever told you that you have any of the following conditions?’’ “Yes” responses were considered to have the condition, while “no” or missing responses were treated as “No.” Questions on frequency and quantity of alcohol use included “How often did you have a drink containing alcohol in the past year?” and “How many drinks did you have on a typical day when you were drinking in the past year?” Cigarette use was based on the number of days the respondent smoked and the number of cigarettes per day. Socio-economic factors included – educational attainment (high school or below, some college or associate’s degree, bachelor’s degree, graduate or professional degree) and household income.

### Statistical analyses

#### Modeling nonresponse

Multinomial logistic regression was used to identify typologies of respondents with different response patterns and to examine demographic and military service characteristics associated with responses across all four waves. Both unadjusted and adjusted odds ratios (ORs) with 95% confidence intervals (CIs) were estimated, using always responders as the reference category. To understand differences between Wave 4 responders and nonresponders, response rates were compared by demographic and military service characteristics using chi-square test measures of association.

To identify and adjust for nonresponse bias at Wave 4, classification and regression tree (CART) analysis was conducted using the SAS^®^ HPSPLIT procedure. CART allows for a multivariate analysis of nonresponse, capturing interactions between the predictor variables and dividing the sample into groups based on response rate. It is easy to interpret and visualize, and the resulting groups can be used as cells in weighting adjustments to reduce bias. A classification tree was generated with response status as the dependent variable and the frame variables as predictors. The default method in HPSPLIT was applied, which employs a purity measure to divide the eligible sample into groups based on response rate, where the predictor variables form the groups. To prevent over-fitting, the tree was pruned by identifying the subtree that minimized cost-complexity, defined as a function of the misclassification rate (cost) and the size of the tree (complexity) [16].

#### Development of weights

The analysis used base weights that allow inference from the stratified sample of 30,000 veterans to the broader population of Gulf War veterans and Gulf Era veterans. These base weights accounted for differential sampling rates by strata and were calculated as the inverse of the selection probability. Using groups identified from the classification tree, respondents were weighted to represent nonrespondents within the same group, thus aligning the weighted respondent distribution with that of the eligible sample (nonresponse adjustment weight). Post-stratification was then applied to adjust the survey weights of respondents so that the weighted sample distribution matched the known population distribution for branch, unit component, deployment status, and sex. The final weights were applied to the 12,377 veterans who completed Wave 4 (final weight).

The effectiveness of the weighting in reducing nonresponse bias was evaluated in four ways. First, estimates of frame variables for the eligible sample were compared to those for respondents weighted by the base weights, nonresponse adjustment weights, and final weights. The first comparison provided a way of assessing if the characteristics of respondents differed from the full eligible sample, that is, if there was nonresponse bias in the estimates of frame variables prior to weighting adjustments. The other comparisons indicated whether the weighting adjustments were effective in reducing the bias. T-tests with adjustments for multiple comparisons were used along with calculations of percentage reduction in bias to determine how much the bias was reduced between the estimates weighted by the base weights and the final weights. The percentage reduction in bias is calculated as the absolute value of the bias before any weighting adjustment minus the absolute value of the bias after the final adjustment, divided by the absolute value of the bias before any weighting adjustment and multiplied by 100. The absolute value of the bias before weighting is the difference in the estimate weighted by the base weights and the eligible sample. The absolute value of the bias after weighting is the difference in the estimate weighted by the final weights and the eligible sample.

Second, the strength of the relationship between frame variables and health outcomes at Wave 4 was measured. The effectiveness of nonresponse bias adjustment depends on how strongly the characteristics used in the adjustment are correlated with survey outcomes [17]. For each health outcome, a logistic regression was fit using nonresponse adjustment cells and post-stratification cells as predictors. A proxy correlation, defined as the square root of the pseudo-R-squared was calculated; values closer to 1 indicate a stronger relationship and greater effectiveness in reducing potential bias in the health outcome.

Third, estimates of the health outcomes were compared before and after weighting adjustments. Changes after weighting suggest that the adjustments reduced nonresponse bias, although the actual magnitude of the existing bias prior to and after nonresponse adjustment is unknown.

Finally, a level-of-effort analysis was conducted to evaluate the potential for nonresponse bias in health outcomes. This approach assumes that late responders are similar to nonrespondents, and so any differences between the late and early respondents would be an indication of nonresponse bias [18–20]. For this purpose, “early responders” were defined to include the first 20% of veterans who completed the survey (among those with ≥ 80% of the question items answered). The “late” responders are the 20% of veterans who responded at the end of the field period. Logistic regression models, using final weights, were used to predict health outcomes. Predictors in the regressions included the indicator for “late” responder and demographic and military service characteristics.

## Results

### Patterns of response over time by group

Of the total Wave 4–eligible sample (*n* = 26,468), there were 4,671 (17.7%) always responders, 7,706 (29.1%) current responders, 9,516 (36.0%) past responders, and 4,575 (17.3%) never responders. After adjustment, the never responder and the past responder groups differed most from the always responder group (Table 1). Predictors of membership in the never responder group included not being deployed, being male, younger age, being Black, Hispanic, or other race/ethnicity, being enlisted, single/other marital status, being in the Navy, and being in the National Guard or reserve. The same variables, except for being in the National Guard/reserve and being male, predicted membership in the past responder group. In general, odds ratios were farther from 1 for those who never responded and past responders compared to current responders. This indicates that those who never responded and past responders were more different from those who always responded (the reference group), and that current responders were more similar to those who always responded. Similarly, many of the ORs for the past responders were farther from 1 than those for the current responders. For example, the adjusted OR for deployment for never responders is 0.319 (0.286–0.356), compared with 0.583 (0.532–0.638) for past responders and 0.687 (0.624–0.755) for current responders. This suggests an ordering in which never responders were least similar to always responders, followed by past responders, with current responders most similar. (Supplementary Table 1 provides the number of veterans in each response group by predictor and Supplementary Table 2 provides crude odds ratios.)

**Table 1:** Adjusted odds ratios for never, past, and current responders relative to always responders

	Never responder		Past responder		Current responder	
	AOR	CI	AOR	CI	AOR	CI
Deployment status						
Not deployed	1.00		1.00		1.00	
Deployed	0.32	0.29–0.36	0.58	0.53–0.64	0.69	0.62–0.76
Sex						
Male	1.00		1.00		1.00	
Female	0.72	0.63–0.82	0.99	0.89–1.09	0.81	0.73–0.89
Race/ethnicity						
White	1.00		1.00		1.00	
Black	4.50	3.86–5.25	2.12	1.83–2.47	2.26	1.95–2.63
Hispanic	1.57	1.22–2.01	1.21	0.97–1.50	1.16	0.91–1.48
Other/unknown	2.60	1.91–3.53	2.15	1.62–2.86	1.78	1.33–2.37
Age in 1991						
17–25	1.00		1.00		1.00	
26–32	0.52	0.45–0.60	0.65	0.57–0.75	0.72	0.63–0.83
33–39	0.23	0.18–0.28	0.37	0.31–0.43	0.52	0.44–0.61
40 and older/unknown	0.13	0.10–0.17	0.26	0.22–0.32	0.34	0.28–0.41
Marital status						
Married	1.00		1.00		1.00	
Single	1.40	1.18–1.67	1.24	1.09–1.41	1.03	0.92–1.17
Other/unknown	1.67	1.18–2.36	1.66	1.23–2.24	1.39	1.07–1.82
Branch						
Army	1.00		1.00		1.00	
Air Force	1.01	0.83–1.24	1.12	1.01–1.24	1.14	1.00–1.29
Marine Corps	1.11	0.99–1.24	1.16	1.01–1.32	1.08	0.95–1.22
Navy	1.55	1.30–1.86	1.29	1.11–1.49	1.10	0.96–1.26
Unit component						
Active duty	1.00		1.00		1.00	
National Guard/ reserves	1.13	1.03–1.24	1.07	0.99–1.16	0.98	0.91–1.07
Rank in 1991						
Enlisted	1.00		1.00		1.00	
Officer/warrant officer	0.42	0.34–0.53	0.63	0.55–0.73	0.74	0.65–0.84

*AOR*  Odds ratio, *CI* 95% confidence interval, ORs are adjusted for all other covariates listed in the table

### Wave 4 response by group

Response rates differed across all demographic and military service characteristics (Table 2). Higher response rates were associated with veterans who were deployed, older, male, white, in the Air Force, officers/warrant officers, and married in 1991. Results from the CART analysis (Table 3) revealed 20 groups with different response rates that were used to develop nonresponse adjustments to reduce the potential bias. The analysis indicates the potential for bias in the survey estimates due to differential representation of subgroups within the respondent sample.

**Table 2:** Wave 4 response rates by demographic and military service characteristics

	Total eligible *n* (col %)	Responded *n* (row %)	*p*-value
Total	26,468	12,377 (46.8%)	
Deployment Status			
Deployed	13,675 (51.7%)	7,149 (52.3%)	< 0.0001
Nondeployed	12,793 (48.3%)	5,228 (40.9%)	
Sex			
Female	5,601 (21.2%)	2,472 (44.1%)	< 0.0001
Male	20,867 (78.8%)	9,905 (47.5%)	
Race/ethnicity			
Black	6,007 (22.7%)	2,339 (38.9%)	< 0.0001
Hispanic	1,242 (4.7%)	522 (42.0%)	
White	18,268 (69.0%)	9,139 (50.0%)	
Other	907 (3.4%)	355 (39.1%)	
Unknown	44 (0.2%)	22 (50.0%)	
Age in 1991			
17–25	11,327 (42.8%)	4,286 (37.8%)	< 0.0001
26–32	7,601 (28.7%)	3,552 (46.7%)	
33–39	4,173 (15.8%)	2,403 (57.6%)	
40 and older	3,342 (12.6%)	2,124 (63.6%)	
Unknown	25 (0.1%)	12 (48.0%)	
Marital status in 1991			
Married	12,871 (48.6%)	6,856 (53.3%)	< 0.0001
Single	12,375 (46.8%)	4,888 (39.5%)	
Other	1,191 (4.5%)	617 (51.8%)	
Unknown	31 (0.1%)	16 (51.6%)	
Branch			
Air Force	3,035 (11.5%)	1,623 (53.5%)	< 0.0001
Army	16,801 (63.5%)	7,822 (46.6%)	
Marine Corps	3,111 (11.8%)	1,363 (43.8%)	
Navy	3,521 (13.3%)	1,569 (44.6%)	
Unit component			
Active duty	10,866 (41.1%)	5,002 (46.0%)	0.04
National Guard	6,753 (25.5%)	3,241 (48.0%)	
Reserves	8,849 (33.4%)	4,134 (46.7%)	
Rank in 1991			
Enlisted	22,873 (86.4%)	10,142 (44.3%)	< 0.0001
Officer	3,322 (12.6%)	2,045 (61.6%)	
Warrant	273 (1.0%)	190 (69.6%)	

**Table 3:** Nonresponse adjustment cells and factors

Group No.	Variable 1	Variable 2	Variable 3	Variable 4	Variable 5	Eligible sample size	Response rate
1	Age 17–25 (in 1991)	Not deployed				5,273	31.3
2		Deployed	Married (in 1991)	Black, Hispanic, Other/Unknown		412	41.9
3				White	Air Force	103	63.1
4					Navy	170	46.2
5					Army or Marine Corps	814	52.3
6			Single, other, unknown	Officer		92	64.1
7				Enlisted or Warrant		4,475	40.5
8	Age 26–32	Not deployed	Black or unknown			949	33.0
9			Hispanic, other, white	Officer, Warrant	Other, Single	202	43.6
10					Married	276	62.4
11				Enlisted		2,234	41.9
12		Deployed	Black or other			1,133	44.6
13			Hispanic, unknown, White			2,820	56.0
14	Age 33+	Black and other	Deployed	Marine Corps, Navy		124	45.4
15				Air Force, Army		725	59.2
16			Not deployed			908	44.4
17		Hispanic, unknown, white	Other, Single	Deployed		598	64.2
18				Not deployed	Air Force, Marine Corps	145	54.1
19					Army, Navy	437	45.0
20			Married, unknown			4,578	66.2

### Comparisons between the eligible sample and responders

In the comparison of eligibles versus responders with base weights, there were differences in marital status, deployment status, rank, and age (Table 4). This suggests that prior to nonresponse adjustment, the characteristics of responders differ from the full eligible sample after accounting for sample design. After applying final weights with nonresponse adjustment, all differences between the eligible sample estimates and final estimates were under 2% points (or under 0.2 for mean age). Bias was reduced for all but two estimates—other and unknown marital status. For these two estimates, the bias in the final estimates was close to zero.

**Table 4:** Comparison of estimates of demographic and military service characteristics with different weights

Frame variable	Value	Eligible sample estimates (1)		Base weighted estimates (2)		*p*-value (2) vs. (1)	NR adjustment weighted estimates (3)		*p*-value (3) vs. (1)	Final weighted estimates (4)		*p*-value (4) vs. (1)	% reduction in bias
		%	Standard error	%	Standard error		%	Standard error		%	Standard error		
Branch of service	Air Force	11.8	0.09	13.4	0.32	< 0.001	12.3	0.27	0.05	11.8	0.09	0.04	96.5
	Army	51.4	0.13	52.2	0.50	0.06	52.7	0.52	0.008	51.5	0.15	0.05	86.8
	Marine	15.2	0.08	14.4	0.31	0.004	15.2	0.31	1.0	15.2	0.08	0.7	98.7
	Navy	21.6	0.11	20.0	0.50	< 0.001	19.8	0.55	< 0.001	21.4	0.16	< 0.001	88.8
Type of service	Active	78.2	0.08	78.5	0.28	0.2	78.7	0.29	0.06	78.2	0.10	0.5	87.5
	Guard	7.6	0.04	7.7	0.13	0.5	7.4	0.14	0.2	7.6	0.05	0.3	69.9
	Reserve	14.2	0.06	13.8	0.22	0.05	13.9	0.24	0.2	14.2	0.08	0.8	96.2
Sex	Male	89.3	0.05	90.3	0.19	< 0.001	89.5	0.24	0.5	89.3	0.06	0.8	99.4
	Female	10.7	0.05	9.7	0.19	< 0.001	10.5	0.24	0.5	10.7	0.06	0.8	99.4
Deployment Status	Deployed	48.7	0.26	53.7	0.50	< 0.001	49.1	0.30	0.001	48.6	0.34	0.8	98.9
	Non-deployed	51.3	0.26	46.3	0.50	< 0.001	50.9	0.30	0.001	51.4	0.34	0.8	98.9
Rank in 1991	Enlisted	86.4	0.29	81.6	0.51	< 0.001	85.3	0.38	< 0.001	85.2	0.38	< 0.001	75.5
	Officer	12.4	0.27	16.5	0.47	< 0.001	13.3	0.37	< 0.001	13.4	0.36	< 0.001	75.9
	Warrant	1.2	0.10	1.8	0.19	< 0.001	1.4	0.14	0.009	1.4	0.14	0.02	72.7
Age group in 1991	17–25	45.6	0.46	37.3	0.62	< 0.001	45.6	0.46	0.8	45.6	0.45	1.0	99.9
	26–32	29.4	0.37	30.1	0.54	0.2	29.4	0.37	0.8	29.4	0.37	0.2	88.3
	33–39	16.0	0.33	20.3	0.53	< 0.001	15.7	0.34	0.03	15.7	0.34	0.03	92.3
	40 +	9.0	0.18	12.4	0.29	< 0.001	9.3	0.23	0.03	9.4	0.24	0.008	88.0
Age in 1991	means	28.2	0.06	29.7	0.09	< 0.001	28.3	0.07	< 0.001	28.3	0.07	< 0.001	93.3
Race/Ethnicity	Black	22.1	0.30	18.1	0.46	< 0.001	20.7	0.44	< 0.001	20.5	0.44	< 0.001	60.8
	Hispanic	4.9	0.19	4.6	0.24	0.09	4.7	0.26	0.4	4.7	0.26	0.3	35.5
	Other	4.0	0.15	3.4	0.25	0.002	3.8	0.24	0.2	3.8	0.25	0.2	60.2
	Unknown	0.1	0.02	0.1	0.03	0.9	0.1	0.03	0.8	0.1	0.03	0.9	47.2
	White	68.9	0.34	73.7	0.47	< 0.001	70.8	0.42	< 0.001	70.9	0.42	< 0.001	59.2
Marital status in 1991	Married	52.8	0.38	60.2	0.60	< 0.001	53.8	0.60	0.02	53.6	0.59	0.07	89.8
	Other	3.3	0.14	3.3	0.19	0.7	3.2	0.17	0.7	3.2	0.17	0.5	-44.4
	Single	43.9	0.37	36.5	0.62	< 0.001	42.9	0.62	0.03	43.2	0.61	0.1	91.1
	Unknown	0.1	0.01	0.1	0.02	0.5	0.1	0.02	0.7	0.1	0.02	0.4	-3.2

### Analysis of health outcomes and sociodemographic characteristics measured in the survey

In general, the correlations between frame variables and health outcomes or sociodemographic characteristics are low to modest, with the strongest correlations for PTSD, GWI, and education (Table 5). The weighting adjustments are expected to be most effective in removing potential bias for variables with higher correlations. Low correlations indicate that the weighting adjustments have limited ability to reduce bias, should it be present.

**Table 5:** Proxy correlations between health outcomes and demographic and military service characteristics used in nonresponse adjustments

Outcome or characteristic	Pseudo *R*-squared	Proxy correlation
General health	0.08	0.28
PTSD	0.11	0.33
GWI	0.10	0.32
# days smoke	0.01	0.11
# cigarettes smoked	0.01	0.11
Alcohol use frequency	0.03	0.17
Alcohol use quantity	0.04	0.21
Alcohol/drug dependence	0.03	0.18
Bipolar disorder or manic depression	0.03	0.16
Education	0.11	0.33
Household income	0.06	0.25

Although differences were observed in some health outcomes and sociodemographic characteristics before and after weighting adjustments, all differences are under 2% points (Table 6). The largest change occurred among respondents with a graduate or professional degree, with a base-weighted estimate of 23.0% compared to the final estimate of 21.3%. The change is in the expected direction, given the weighting adjustments correct for the over-representation of officers among responders.

**Table 6:** Estimates of health outcomes and sociodemographic characteristics (%) with different weights

Outcome or characteristic		Base-weighted estimates (1)	NR adjustment weighted estimates (2)	*p*-value (2) vs. (1)	Final Weighted Estimates (3)	*p*-value (3) vs. (2)
General health	Excellent	3.9	3.7	< 0.001	3.7	0.001
	Very good	18.6	18.1	< 0.001	18.2	0.06
	Good	38.4	38.9	0.001	38.9	0.8
	Fair	31.5	31.8	0.05	31.7	0.05
	Poor	7.6	7.6	0.8	7.6	0.01
PTSD	Yes	29.6	30.7	< 0.001	30.3	< 0.001
GWI	Yes	12.1	11.7	< 0.001	11.5	< 0.001
Alcohol or drug dependence	Yes	10.1	10.6	< 0.001	10.6	0.4
Bipolar disorder or manic depression	Yes	6.2	6.5	< 0.001	6.5	0.3
Alzheimer’s disease or dementia	Yes	1.7	1.5	< 0.001	1.5	0.1
Smoking frequency	0 days smoked	89.4	89.2	0.03	89.2	0.6
	1 day smoked	#	0.1	0.7	#	0.3
	2 days smoked	0.2	0.2	0.05	0.2	0.4
	3 to 7 days smoked	0.4	0.4	0.8	0.4	0.7
	8 to 14 days smoked	0.4	0.4	0.03	0.4	0.2
	15 to 29 days smoked	1.5	1.6	0.06	1.6	0.7
	30 days smoked	8.1	8.1	0.5	8.1	0.2
Smoking quantity	Did not smoke in the past 30	89.6	89.4	0.03	89.4	0.5
	1 or 2 cigarettes per day	0.8	0.9	0.06	0.9	1.0
	3 to 10 cigarettes per day	4.0	4.2	0.06	4.2	0.7
	11 to 20 cigarettes per day	4.1	4.1	0.3	4.1	0.1
	21 or more cigarettes per day	1.4	1.4	1.0	1.4	0.5
Alcohol use frequency	Never	25.6	24.9	< 0.001	24.9	1.0
	Monthly or less	25.2	25.6	0.03	25.5	0.7
	Two to four times a month	17.2	17.4	0.02	17.5	0.2
	Two to three times a week	14.9	15.1	0.07	15.1	0.8
	Four or more times a week	17.1	17.0	0.7	17.0	0.8
Alcohol use quantity	None	28.5	27.7	< 0.001	27.7	1.0
	1 or 2 drinks	47.1	46.6	0.003	46.5	0.7
	3 or 4 drinks	15.7	16.4	< 0.001	16.4	0.2
	5 or 6 drinks	5.4	5.7	< 0.001	5.7	0.7
	7 to 9 drinks	1.8	2.0	< 0.001	2.0	0.5
	10 or more drinks	1.5	1.7	0.001	1.6	0.07
Educational attainment	High school or below	14.9	15.5	< 0.001	15.5	0.5
	Some college or associates	41.9	42.8	< 0.001	42.7	0.2
	Bachelor’s degree	20.3	20.5	0.1	20.5	0.9
	Graduate or professional degree	23.0	21.3	< 0.001	21.3	0.5
Household income	0–34,999	9.6	9.8	0.01	9.9	0.2
	35,000–49,999	10.1	10.0	0.04	10.0	0.4
	50,000–74,999	20.1	20.0	0.5	20.0	0.8
	75,000–99,999	18.4	18.2	0.05	18.1	0.2
	100,000+	41.8	42.0	0.2	42.1	0.4

(#) Rounds to zero

### Analysis of early versus late responders

Several estimates of physical and mental health differed between the early and late responders (Table 7). This analysis assumes that late responders are more similar to nonrespondents than early responders; thus, differences may indicate potential nonresponse bias. If late responders and nonrespondents are different, using late responders as a proxy for nonrespondents may be unreliable. Late responders were more likely than early responders to report alcohol or drug dependence, daily smoking, smoking more than half a pack per day, and having seven or more drinks per day. Odds ratios for poor health, bipolar disorder/manic depression, Alzheimer’s disease/dementia, and drinking for or more times per week were also elevated. (Supplementary Table 3 provides the number of early and late responders by each health outcome and sociodemographic characteristic.)

**Table 7:** Adjusted odds ratios for late versus early responders on health outcomes and sociodemographic characteristics

Health Outcome or Sociodemographic Characteristics		AOR (Late versus early)	CI	*p*-value
General health status	Poor	1.64	0.98–2.72	0.06
	Fair to excellent	1.00		
PTSD	Yes	1.02	0.83–1.25	0.8
	No	1.00		
GWI	Yes	0.82	0.61–1.11	0.2
	No	1.00		
Past month smoking frequency	Daily	1.87	1.41–2.47	< 0.0001
	Less than daily	1.00		
Past month smoking quantity	10 or more cigarettes daily	1.79	1.25–2.57	0.002
	Fewer than 10 cigarettes daily	1.00		
Past year alcohol frequency	4 or more times per week	1.25	0.99–1.58	0.06
	Fewer than 4 times per week	1.00		
Drinks per occasion	7 or more drinks	1.69	1.05–2.37	0.03
	Fewer than 7 drinks	1.00		
Alcohol/drug dependence	Yes	1.54	1.13–2.10	0.007
	No	1.00		
Bipolar or manic depression	Yes	1.44	0.99–2.11	0.06
	No	1.00		
Alzheimer’s disease or dementia	Yes	1.58	0.83–3.01	0.2
	No	1.00		
Education	Graduate or professional degree	0.59	0.42–0.82	0.002
	Bachelor’s degree or lower	1.00		
Household income	$100,000 or more	0.53	0.38–0.74	0.0003
	Less than $100,000	1.00		

ORs adjusted for age, sex, rank, unit component, deployment status, branch, race/ethnicity, and marital status in 1991

*AOR*  Adjusted odds ratio, *CI* 95% confidence interval

## Discussion

GWECS is the largest and longest-running longitudinal cohort study of Gulf War veterans. It has contributed much of what is known about the health effects of deployment to the Gulf and has resulted in more than 30 peer-reviewed publications. Wave 4 obtained a response rate of 47%, which is very close to the 50% response rate obtained in Wave 3 over a decade earlier. As such, Wave 4 was a success.

Wave 4 of the study encountered a differential response similar to that observed in other longitudinal studies of the general population and veterans. Three groups of veterans with a history of nonresponse differed from those who responded to all four waves of the study. Many of the factors associated with never responding were also associated with inconsistent response across survey waves including non-deployed, enlisted, younger age, Black/Hispanic/other race/ethnicity, and single/other marital status in 1991. However, nonresponse was more a matter of degree than kind, with those who never responded being the most different on the characteristics examined compared to those who always responded followed by past responders and current responders.

Weighting adjustments effectively accounted for differences and reduced bias in demographic and military characteristics. Estimates of health outcomes were similar before and after weighting adjustment. Given the modest correlations between health outcomes and the demographic and military characteristics used in weighting, weighting is likely to have had limited impact on possible bias in health outcomes. Because there are no benchmarks for health outcomes for a unique survey like GWECS, we compared several health outcomes between early and late responders. Estimates of alcohol or drug dependence, as well as alcohol use and smoking, were higher among the late responders. Assuming that late responders are similar to nonresponders, this could indicate possible bias in these estimates.

Several limitations to the analysis should be noted. First, reasons for nonresponse were not distinguished, such as inability to contact (e.g., did not receive the mailed invitation because of a change of address) or refusal (e.g., received the mailed invitation but chose not to respond). Veterans who refused to participate may have different demographic and military service characteristics than those who could not be contacted. Second, individual response patterns related to survey responses on physical and mental health at Wave 1 could not be examined because not all veterans completed the Wave 1 survey. Future analyses should focus on those who participated in Wave 1 and model their future participation typologies based on baseline health indicators. In addition, because physical and mental health are likely to change over time, examining how these factors at each wave impact subsequent response could yield important insights. Finally, possible bias in self-reported health outcomes may make it difficult to disentangle the effects of nonresponse bias from measurement error.

The findings suggest the importance of continuing to exert effort into maintaining participation among veterans whose characteristics are associated with nonresponse. One of the factors most strongly associated with response over time was being deployed. This may be because deployed veterans have greater health concerns, which could increase their interest in participating in an epidemiological study. Deployment may also have contributed to stronger identification with the cohort, further encouraging participation. For those who were not deployed, it will be important to continue to emphasize why participation matters for the study as a whole. Future cohort studies may also wish to conduct targeted oversampling of groups that are likely to be underrepresented. While nonresponse adjustments can mitigate bias, they are not a replacement for collected data, especially when performing stratified analysis. GWECS and other epidemiological studies of veterans will need to continue to focus on efforts to increase participation among underrepresented groups.

It has now been over 30 years since the 1990–1991 Gulf War, yet many concerns and unknown questions remain about the long-term health effects of service in the Gulf War. The aging of the Gulf War veteran population confounds many of these issues, as almost all cancers and chronic diseases are associated with aging making it difficult to untangle age-related morbidity from deployment-related exposures. There are many questions surrounding the health of women Gulf War veterans, an understudied population, as they age. As the only study following a population-based cohort that includes a 20% sample of women Gulf War and Gulf War Era veterans, GWECS has contributed greatly to the literature in this area [21]. Overall, GWECS is an invaluable resource for the study of the health effects of deployment-related toxic exposures, the health effects of military service, and the gender specific health effects.

## Conclusion

Despite declining survey response rates, [22] GWECS successfully maintained its response rate after a decade. Findings on the demographic and military service characteristics associated with response patterns over time provide useful information on groups that may require additional effort and resources to encourage survey completion. While the use of nonresponse weights reduced bias in demographic and military service characteristics, the analysis showed that late responders were more likely than early responders to report alcohol or drug dependence, daily smoking, smoking more than half a pack per day, and having seven or more drinks per day. These results are consistent with other longitudinal surveys where nonresponders and late responders tend to engage in less healthy lifestyles [23] and riskier health behaviors than responders. This includes an increased risk of alcohol-, smoking-, and drug-related mortality and morbidity [24]. Assuming that late GWECS responders are more similar to nonresponders than responders, these findings suggest that even with the nonresponse adjustment, it is possible that the levels of alcohol and drug dependence may be underestimated. These findings may be useful to other studies of veterans as well as general population studies.

## Supplementary Information

Supplementary Material 1.

## Data Availability

The datasets generated and/or analyzed during the current study are not publicly available because the consent states that the data will only be available to the VA study team.

## Abbreviations

GWECS: Gulf War Era Cohort Study
VA: U.S. Department of Veterans Affairs
MCS: Millennium Cohort Study
DMDC: U.S. Department of Defense’s Defense Manpower Data Center
CATI: Computer-assisted telephone interviewing
CART: Classification and regression tree
ORs: Odds ratios
CIs: Confidence intervals
PTSD: Posttraumatic stress disorder
GWI: Gulf War Illness
