# Supplementary material for: Assessing nonresponse bias in a 30-year study of gulf war and gulf era veterans
Source: BMC Med Res Methodol. 2026 Jan 10;26:27. doi: 10.1186/s12874-025-02761-5 (PMC12882365; doi:10.1186/s12874-025-02761-5)
Supplement: Supplementary file 1 — Supplementary Material 1. [file 12874_2025_2761_MOESM1_ESM.docx]

Supplementary Table 1. Number of always, never, past, and current responders

|  | Total | Always responder | Never responder | Past responder | Current responder |
| --- | --- | --- | --- | --- | --- |
| Deployment status | | | | | |
| Not deployed | 12,793 | 1,714 | 2,891 | 4,674 | 3,514 |
| Deployed | 13,675 | 2,957 | 1,684 | 4,842 | 4,192 |
| Sex | | | | | |
| Male | 20,867 | 3,716 | 3,631 | 7,331 | 6,189 |
| Female | 5,601 | 955 | 944 | 2,185 | 1,517 |
| Race/ethnicity | | | | | |
| White | 18,268 | 3,841 | 2,537 | 6,592 | 5,298 |
| Black | 6,007 | 557 | 1,586 | 2,082 | 1,782 |
| Hispanic | 1,242 | 163 | 254 | 466 | 359 |
| Other/unknown | 951 | 110 | 198 | 376 | 267 |
| Age in 1991 | | | | | |
| 17–25 | 11,327 | 1,243 | 2,584 | 4,457 | 3,043 |
| 26–32 | 7,601 | 1,248 | 1,285 | 2,764 | 2,304 |
| 33–39 | 4,173 | 1,005 | 464 | 1,306 | 1,398 |
| 40 and older/unknown | 3,367 | 1,175 | 242 | 989 | 961 |
| Marital status | | | | | |
| Married | 12,871 | 1,704 | 2,843 | 4,311 | 4,013 |
| Single | 12,375 | 1,575 | 2,714 | 4,773 | 3,313 |
| Other/unknown | 1,222 | 2,53 | 157 | 432 | 380 |
| Branch | | | | | |
| Army | 16,801 | 2,945 | 2,944 | 6,035 | 4,877 |
| Air Force | 3,035 | 643 | 374 | 1,038 | 980 |
| Marine Corps | 3,111 | 470 | 577 | 1,171 | 893 |
| Navy | 3,521 | 613 | 680 | 1,272 | 956 |
| Unit component | | | | | |
| Active duty | 10,866 | 1,740 | 1,956 | 3,908 | 3,262 |
| National Guard/ reserves | 15,602 | 2,931 | 2,619 | 5,608 | 4,444 |
| Rank in 1991 | | | | | |
| Enlisted | 22,873 | 3,552 | 4,284 | 8,447 | 6,590 |
| Officer/warrant officer | 3,595 | 1,119 | 291 | 1,069 | 1,116 |

Supplementary Table 2. Unadjusted odds ratios for never, past, and current responders relative to always responders

|  | Never responder | | Past responder | | Current responder | |
| --- | --- | --- | --- | --- | --- | --- |
|  | **OR** | **CI** | **OR** | **CI** | **OR** | **CI** |
| Deployment status | | | | | | |
| Not deployed | 1.00 |  | 1.00 |  | 1.00 |  |
| Deployed | 0.43 | 0.39 –0.47 | 0.71 | 0.65–0.77 | 0.81 | 0.74–0.88 |
| Sex | | | | | | |
| Male | 1.00 |  | 1.00 |  | 1.00 |  |
| Female | 1.10 | 0.98–1.23 | 1.21 | 1.11–1.32 | 0.96 | 0.88–1.06 |
| Race/ethnicity | | | | | | |
| White | 1.00 |  | 1.00 |  | 1.00 |  |
| Black | 4.54 | 3.92–5.26 | 2.19 | 1.91–2.52 | 2.31 | 2.00–2.67 |
| Hispanic | 1.79 | 1.41–2.29 | 1.34 | 1.07–1.66 | 1.25 | 0.98–1.60 |
| Other/unknown | 2.37 | 1.76–3.19 | 2.01 | 1.51–2.69 | 1.68 | 1.27–2.23 |
| Age in 1991 | | | | | | |
| 17–25 | 1.00 |  | 1.00 |  | 1.00 |  |
| 26–32 | 0.45 | 0.40 –0.51 | 0.59 | 0.53–0.66 | 0.71 | 0.63–0.80 |
| 33–39 | 0.20 | 0.16–0.24 | 0.33 | 0.28–0.38 | 0.52 | 0.45–0.60 |
| 40 and older/unknown | 0.10 | 0.08–0.12 | 0.22 | 0.19–0.25 | 0.32 | 0.27–0.37 |
| Marital status | | | | | | |
| Married | 1.00 |  | 1.00 |  | 1.00 |  |
| Single | 2.88 | 2.51–3.30 | 2.06 | 1.86–2.29 | 1.49 | 1.35–1.64 |
| Other/unknown | 1.34 | 0.97–1.85 | 1.49 | 1.15–1.94 | 1.27 | 0.99–1.63 |
| Branch | | | | | | |
| Army | 1.00 |  | 1.00 |  | 1.00 |  |
| Air Force | 0.61 | 0.51–0.72 | 0.84 | 0.76–0.94 | 0.92 | 0.83–1.02 |
| Marine Corps | 1.23 | 1.10–1.37 | 1.27 | 1.12–1.44 | 1.15 | 1.03–1.30 |
| Navy | 1.25 | 1.06–1.48 | 1.16 | 1.02–1.33 | 1.01 | 0.89–1.15 |
| Unit component | | | | | | |
| Active duty | 1.00 |  | 1.00 |  | 1.00 |  |
| National Guard/ reserves | 0.96 | 0.88–1.05 | 0.93 | 0.86–1.00 | 0.86 | 0.80–0.92 |
| Rank in 1991 | | | | | | |
| Enlisted | 1.00 |  | 1.00 |  | 1.00 |  |
| Officer/warrant officer | 0.19 | 0.15–0.23 | 0.38 | 0.33–0.43 | 0.50 | 0.44–0.57 |

OR = Odds ratio; CI: 95% confidence interval.

Supplementary Table 3. Number of early and late responders by health outcomes and sociodemographic characteristics

| **Outcome or Characteristic** |  | **Early Responders** | **Late Responders** |
| --- | --- | --- | --- |
| General health status | Poor | 128 | 148 |
|  | Fair to excellent | 2,172 | 2,734 |
| PTSD | Yes | 698 | 851 |
|  | No | 1,576 | 1,668 |
| GWI | Yes | 312 | 380 |
|  | No | 1,989 | 2,114 |
| Past month smoking frequency | Daily | 152 | 254 |
|  | Less than daily | 2,132 | 2,235 |
| Past month smoking quantity | 10 or more cigarettes daily | 98 | 151 |
|  | Fewer than 10 cigarettes daily | 2,284 | 2,487 |
| Past year alcohol frequency | 4 or more times per week | 370 | 367 |
|  | Fewer than 4 times per week | 1,914 | 2,128 |
| Drinks per occasion | 7 or more drinks | 49 | 76 |
|  | Fewer than 7 drinks | 2,234 | 2,414 |
| Alcohol/drug dependence | Yes | 158 | 257 |
|  | No | 2116 | 2,275 |
| Bipolar or manic depression | Yes | 121 | 189 |
|  | No | 2,180 | 2,347 |
| Alzheimer's disease or dementia | Yes | 34 | 58 |
|  | No | 2,267 | 2481 |
| Education | Graduate or professional degree | 584 | 506 |
|  | Bachelor’s degree or lower | 1,688 | 2,004 |
| Household income | $100,000 or more | 984 | 922 |
|  | Less than $100,000 | 1,224 | 1,480 |

PTSD = Posttraumatic stress disorder; GWI = Gulf War Illness
